# Protective Effects of Sitagliptin on Dextran Sulfate Sodium-Induced Colitis via Modulation of Inflammatory and Oxidative Stress

**DOI:** 10.3390/medicina62061005

**Published:** 2026-05-22

**Authors:** Vivian Soetikno, Mawar Subangkit, Andika Yusuf Ramadhan, Somasundaram Arumugam, Remya Sreedhar

**Affiliations:** 1 Department of Pharmacology and Therapeutics, Faculty of Medicine, Universitas Indonesia, Jakarta 10430, Indonesia; 2 School of Veterinary Medicine and Biomedical Sciences, Division of Pathology, Institute Pertanian Bogor University, Bogor 16680, Indonesia; msbangkit@gmail.com; 3 Clinical Pharmacology Specialist Program, Faculty of Medicine, Universitas Indonesia, Jakarta 10430, Indonesia; dr.andikayusufr@gmail.com; 4 Department of Pharmacology & Toxicology, National Institute of Pharmaceutical Education and Research, Kolkata 700054, India; somasundaram143@gmail.com; 5 School of Pharmacy, Sister Nivedita University, Kolkata 700156, India; sreedharremya@gmail.com

**Keywords:** dextran sulfate sodium, ulcerative colitis, oxidative stress, inflammation, sitagliptin

## Abstract

*Background:* To examine the antioxidant and anti-inflammatory effects of sitagliptin in restoring the intestinal mucosal barrier in rats with colitis induced by dextran sulfate sodium (DSS). *Methods:* Male Sprague-Dawley rats were administered 5% DSS in their drinking water to induce colitis. Sitagliptin was administered intragastrically at a dose of 15 mg/kg/day for a duration of eight days. Changes in the colon tissue were histologically examined, and the disease activity index (DAI) score was measured. The levels of malondialdehyde (MDA), superoxide dismutase (SOD), glutathione peroxidase (GPx), and catalase were evaluated. Gene expression of tumor necrosis factor (TNF)-α, interleukin (IL)-1β, tight junction proteins occludin and ZO-1 was assessed. Levels of SGOT, SGPT, and serum iron were also measured. *Results:* Sitagliptin diminished DAI and histological index scores, as well as MDA levels, while augmenting SOD, GPx, and catalase levels over an eight-day period. Based on proinflammatory cytokines, sitagliptin reduced colon inflammation. Compared to the untreated DSS group, sitagliptin increased serum iron and lowered SGOT and SGPT. *Conclusions:* The present results indicate that administering sitagliptin orally for a week could aid in the recovery from DSS-induced colitis by reducing oxidative stress and pro-inflammatory cytokines. Additional studies are required to make this applicable for patients suffering from colitis.

## 1. Introduction

Ulcerative colitis (UC) is a chronic and debilitating inflammatory bowel disease (IBD) characterized by damage to the intestinal barrier and inflammatory issues, primarily affecting the colon and rectum [1]. The morbidity of UC in East Asia shows an increasing trend in IBD cases in general, although the specific incidence of UC is still low, at around 2.2–14.3 cases per 100,000 population in 2021. Generally, the symptoms experienced are mild to moderate, but this disease is chronic and can cause long-term complications if not treated properly [2,3]. At present, there are limited clinical treatment options, mainly including 5-aminosalicylates, corticosteroids, immunosuppressive agents, and TNF-α inhibitors [4]. The insufficient efficacy and notable side effects of existing treatments highlight the urgent need for new methods to address UC, with drug repurposing emerging as a promising approach that investigates the possibility of using established medications with recognized safety records for alternative therapeutic purposes.

The exact cause of UC has not been determined; however, it is believed to result from a combination of genetic factors, environmental elements like smoking, diet, infections, certain medications, and an abnormal immune reaction. This leads to chronic inflammation and increased oxidative stress, causing the immune system to mistakenly attack the colon, resulting in inflammation of the lining, the development of ulcers, and elevated permeability of the colon mucosa due to the downregulation of tight-junction proteins like ZO-1 and occludin [5]. Recently, increasing evidence has suggested the involvement of iron dysregulation and ferroptosis-related oxidative injury in experimental colitis, particularly in relation to impaired antioxidant defenses and excessive lipid peroxidation [6,7]. Furthermore, extraintestinal manifestations of UC have also been reported, including in the liver [8].

Sitagliptin is a DPP-4 inhibitor, commonly used as a treatment for type 2 diabetes mellitus due to its ability to prevent the breakdown of GLP-1 [9]. A previous study by Ning et al. reported that there was an increase in DPP-4 enzyme activity in colon tissue of the dextran sulfate sodium (DSS)-induced colitis model and that sitagliptin administration could prevent apoptosis and increase proliferation of intestinal barrier epithelium in colitis through the increase of GLP-2 peptide hormone activity [10]. Sitagliptin has been involved in various processes such as oxidative stress, inflammation, apoptosis signaling pathways, regulation of the immune response, and glucose metabolism [11,12]. However, the relationship between sitagliptin, iron dysregulation, ferroptosis-related oxidative injury, and intestinal barrier integrity in DSS-induced UC remains insufficiently explored.

Therefore, the current research focused on investigating the repurposing of sitagliptin by evaluating oxidative stress, inflammatory responses, intestinal barrier integrity, and iron alterations associated with ferroptosis-related oxidative injury in DSS-induced colitis.

## 2. Materials and Methods

### 2.1. Reagents

Sitagliptin (Cat. No. 654671-77-99) and dextran sulfate sodium (Cat. No. 67578) were obtained from Sigma-Aldrich Co. (St. Louis, MO, USA). Rat GPx (Cat. No. MBS744364), SOD (Cat. No. MBS036924), and catalase (Cat. No. MBS2704433) ELISA kits were purchased from MyBioSource (San Diego, CA, USA). Kits for detecting SGPT (Cat. No. 1 2701 99 10 021), SGOT (Cat. No. 1 2601 99 83 021), and iron (Fe) (Cat. No. 1 1911 99 83 021) were purchased from DiaSys Diagnostic Systems GmbH, Holzheim, Germany. Trichloroacetic acid (Cat. No. 1.00807.0250) and 2-thiobarbituric acid (Cat. No. 1.08180.0025) were acquired from Merck (Darmstadt, Germany), while 1,1,3,3-tetramethoxy propane (Cat. No. T-1642) was sourced from Sigma-Aldrich (St. Louis, MO, USA). TRIzol was sourced from Thermo Fisher Scientific (Waltham, MA, USA). The Prime Script RT Reagent Kit along with the SYBR Premix Ex Taq Kit, was obtained from Takara Biomedical Technology (Dalian, China).

### 2.2. Animals

Eighteen male Sprague-Dawley rats (aged 6–8 weeks and weighing between 150–200 g) were obtained from the National Agency of Food and Drug Control in Indonesia. The animals were kept on a 12-h light and 12-h dark cycle, with unrestricted access to standard chow and water. The Institutional Animal Care and Use Committee of the Faculty of Medicine at Universitas Indonesia approved the animal experiments (KET-1234/UN2.F1/ETIK/PPM.00.02/2025). A total of eighteen animals were randomly allocated into three groups, each consisting of six animals, categorized as follows: Group 1: Normal control; Group 2: DSS control; and Group 3: DSS + Sitagliptin (15 mg/kg/day). The basis for determining the sitagliptin dose used in this study is the conversion of the sitagliptin dose used in humans (100 mg/day) using the body surface area formula [13]. Every animal, apart from the standard control group, received 5% DSS in their drinking water from day 0 through day 7 [14,15]. Sitagliptin was given orally to rats from day 0 through day 8. The disease activity index (DAI) was assessed daily. All animals were euthanized on day 8 through an overdose of ketamine/xylazine injection (three times the anesthetic dose of ketamine and xylazine, namely 300 and 6 mg/kg). Blood was collected via aortic puncture, and colon samples were obtained for subsequent analysis. All procedures adhered to the ARRIVE guidelines to reduce suffering in the animals and complied with the principles of the 3Rs (Replacement, Reduction, and Refinement) to ensure ethical and responsible use of experimental animals. Animals were randomly allocated into experimental groups using a simple randomization method. In addition, histopathological analysis was performed in a blinded manner to reduce observer bias during data assessment.

### 2.3. Evaluation of the DAI

The DAI was assessed as previously outlined by scoring body weight loss (0 points, <1%; 1 point, 1–5%; 2 points, 5–10%; 3 points, 10–20%; and 4 points, >20%), stool consistency (0 points, well-formed feces; 2 points, semiformed stools; and 4 points, diarrhea), and fecal occult blood (0 points, negative; 2 points, positive; and 4 points, gross bleeding) [16].

### 2.4. Colon Collection and Measurement

Colon sections from rats were carefully removed, and their lengths were recorded. After eliminating feces, weight measurements were taken. The colons were then washed with cold saline solution. The area near the anus was fixed in a 10%, pH 7.4 buffered formalin for more than 24 h, embedded in paraffin, cut into 4 µm thick sections, and stained with hematoxylin and eosin for examination under a light microscope. The remaining colon samples were snap-frozen and stored at −80 °C for further examination. Two pathologists, who were unaware of the treatment details, evaluated each colon specimen based on previously established criteria for two parameters: the infiltration of inflammatory cells (0 points for no inflammatory infiltration; 1 point for minimal infiltration in the lamina propria; 2 points for high levels of inflammatory cells in the lamina propria; and 3 points for infiltration of inflammatory cells into the submucosa) and tissue damage (0 points indicating no damage; 1 point for minor epithelial lesions; 2 points for moderate damage to the mucous membrane and localized ulcers; and 3 points for extensive destruction of the mucosal structure extending to the muscle layer of the intestine) [17].

### 2.5. Blood and Serum Analysis

At the conclusion of the experiment on day 8, blood samples were collected via aortic puncture while the subjects were under anesthesia. The blood was left to clot at room temperature before being centrifuged at 3000 rpm for 10 min to obtain serum. The isolated serum was then utilized for biochemical analysis. The serum iron concentration was measured using the colorimetric Ferrozine method with a commercial kit supplied by DiaSys Diagnostic Systems GmbH (Holzheim, Germany), with absorbance readings taken at 560 nm on a UV-Vis spectrophotometer (Shimadzu, Japan). Serum alanine aminotransferase (SGPT) and aspartate aminotransferase (SGOT) activities were assessed using the kinetic UV method with DiaSys reagent kits, with absorbance monitored at 340 nm as per the manufacturer’s guidelines.

### 2.6. Determination of Malondialdehyde and Antioxidant Enzymes Levels

About 100 mg of distal colon tissue was removed, rinsed with ice-cold phosphate-buffered saline (PBS, pH 7.4), dried with a blotting paper, and homogenized (10% *w*/*v*) using a glass-Teflon homogenizer in ice-cold 1.15% KCl for the malondialdehyde (MDA) assay or in PBS (pH 7.4) for the antioxidant enzyme assays. The homogenates were subjected to centrifugation at 10,000× *g* for 10 min at 4 °C, and the resulting supernatants were gathered for biochemical analysis.

MDA levels, which serve as an indicator of lipid peroxidation, were assessed using the thiobarbituric acid reactive substances (TBARS) method. In brief, a portion of the supernatant was combined with 10% trichloroacetic acid (TCA), subjected to centrifugation, and the resulting supernatant was treated with 0.67% thiobarbituric acid (TBA) in 20% acetic acid (pH 3.5) at 95 °C for 15 min. After allowing it to cool, the absorbance was measured at 532 nm with a UV-Vis spectrophotometer (Shimadzu, Japan). MDA concentrations were derived from a standard curve of 1,1,3,3-tetramethoxypropane (TMP) and reported as nmol/mg protein.

The levels of antioxidant enzymes such as glutathione peroxidase (GPx), superoxide dismutase (SOD), and catalase were evaluated in the colon supernatant using commercial ELISA kits, following the manufacturer’s instructions. Absorbance was measured at 450 nm using a microplate reader, and enzyme levels were presented as ng/mg protein.

### 2.7. Determination of TNF-α and IL-1β

Total RNA was extracted from colonic tissues using TRIzol. The expression levels of mRNA for specific genes were evaluated with the Prime Script RT Reagent Kit containing gDNA Eraser and the SYBR Premix Ex Taq Kit, and measurements were obtained through the Bio-Rad CFX96 Real-Time PCR Detection Systems (Hercules, CA, USA). The primer sequences used in this research are provided in Table 1. Rat β-actin was utilized as the reference gene.

### 2.8. Statistical Analysis

Statistical evaluations were performed utilizing the GraphPad Prism software version 10.6.0 (GraphPad Software, San Diego, CA, USA). Data are expressed as the mean ± standard deviation (SD). All statistical evaluations were carried out using one-way ANOVA, followed by Tukey’s post-hoc test. Differences between groups were considered significant at *p* < 0.05. Data normality was assessed using the Shapiro-Wilk test prior to statistical analysis.

## 3. Results

### 3.1. Effect of Sitagliptin on Disease Severity in DSS-Induced Colitis Rats

**Figure 1:**
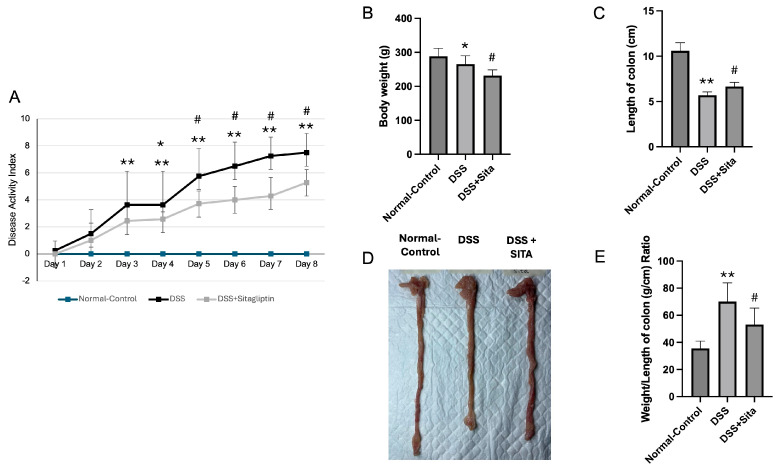
Sitagliptin improved the disease activity index, body weight, length of colon, and weight/length of colon ratio. DSS (5%) was given for seven days to induce experimental colitis in rats. Sitagliptin 15 mg/kg/day or vehicle (distilled water) was given orally to the relevant groups from day 0 to day 8. (**A**) Disease activity index (DAI) score. (**B**) Body weight. (**C**) Colon length. (**D**) Representative images of colons on day 8. (**E**) Colon weight/length ratio. Data are expressed as the mean ± SD (*n* = 6 rats/group). * *p* < 0.05, ** *p* < 0.01 vs. normal-control group; ^#^ *p* < 0.05 vs. DSS group.

To assess the effectiveness of sitagliptin in alleviating the severity of DSS-induced colitis, we evaluated the DAI score and changes in body weight during the study and analyzed the colon length-to-body weight ratio and the macroscopic appearance of the colon. As shown in Figure 1A, administration of 5% DSS solution in drinking water resulted in colitis in rats. DAI scores increased significantly on day 3 after administration of 5% DSS in drinking water, and all rats experienced changes in stool consistency during the study. The occurrence of colitis was characterized by the presence of blood in the stool, diarrhea, and weight loss (Figure 1B). Colitis also caused colon shortening (Figure 1C,D) and an increase in the colon weight/length ratio (Figure 1E). Sitagliptin administration significantly decreased the DAI score from day 3 to day 8 (Figure 1A). Sitagliptin administration also significantly improved the body weight, the length of the colon, and the colon weight/length ratio (Figure 1B–E).

### 3.2. Effect of Sitagliptin on Serum Biochemical Parameters in DSS-Induced Colitis Rats

To determine whether there is extraintestinal organ involvement in UC, we checked serum SGOT and SGPT levels, and we checked serum iron levels to assess iron dysregulation. Serum levels of biochemical enzymes, including SGOT and SGPT, were higher in DSS-induced colitis-induced rats than in normal rats. Additionally, we demonstrated that, in comparison to the normal rats, the DSS-induced colitis rats had considerably lower serum iron levels. The administration of sitagliptin resulted in significantly elevated serum iron levels and reduced SGOT and SGPT levels (Table 2).

### 3.3. Effect of Sitagliptin on Oxidative Damage to Intestinal Mucosa in DSS-Induced Colitis Rats

**Figure 2:**
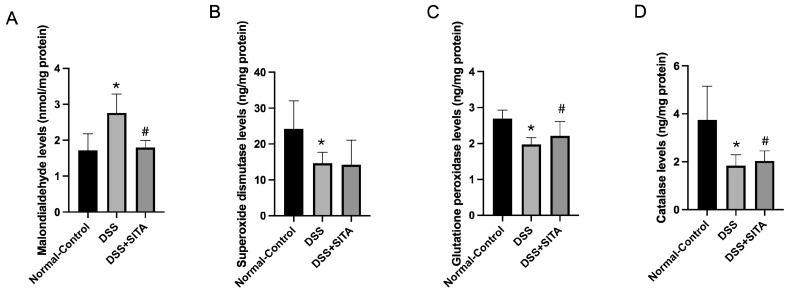
Sitagliptin ameliorated oxidative stress in the colon tissue of DSS-induced colitis rats. (**A**) Malondialdehyde levels. (**B**) Superoxide dismutase levels. (**C**) Glutathione peroxidase levels. (**D**) Catalase levels. Data are expressed as the mean ± SD (*n* = 6 rats/group). * *p* < 0.05 vs. normal-control group; ^#^ *p* < 0.05 vs. DSS group.

Next, we measured MDA levels and antioxidant levels to determine whether oxidative damage was present. In comparison to the normal-control rats, DSS administration elevated MDA levels in the colon while reducing the levels of SOD, GPx, and catalase. The administration of sitagliptin reversed the alterations produced by DSS as previously documented (Figure 2A–D).

### 3.4. Effect of Sitagliptin on Inflammation and on Tight-Junction Components in DSS-Induced Colitis Rats

**Figure 3:**
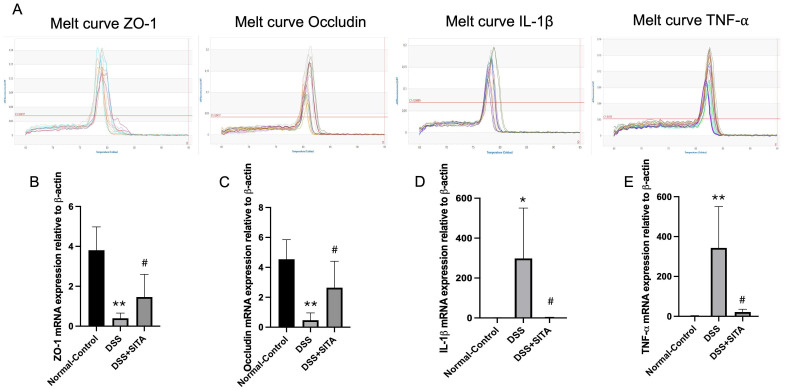
Sitagliptin ameliorated inflammation and tight junction protein in the colon tissue of DSS-induced colitis rats. (**A**) Melting curve of ZO-1, occludin, IL-1β, and TNF-α expression in colon. mRNA expression of ZO-1 (**B**), occludin (**C**), IL-1β (**D**), and TNF-α (**E**). Data are expressed as the mean ± SD (*n* = 6 rats/group). * *p* < 0.05, ** *p* < 0.01 vs. normal-control group; ^#^ *p* < 0.05 vs. DSS group.

To assess the inflammatory process in colonic tissue and the integrity of the epithelial barrier, we analyzed the expression of the IL-1β, TNF-α, ZO-1, and occludin genes. The colon tissues of rats with DSS-induced colitis demonstrated an elevation in proinflammatory mediators and a reduction in tight junction (TJ) components. The DSS group exhibited a considerable decrease in ZO-1 and occludin mRNA levels, as illustrated in Figure 3A–C. In comparison to normal-control rats, the levels of the proinflammatory cytokines IL-1β and TNF-α in DSS-induced colitis rats were significantly elevated, which were diminished by sitagliptin administration (Figure 3A,D,E).

### 3.5. Effect of Sitagliptin on Histopathological Results in DSS-Induced Colitis Rats

**Figure 4:**
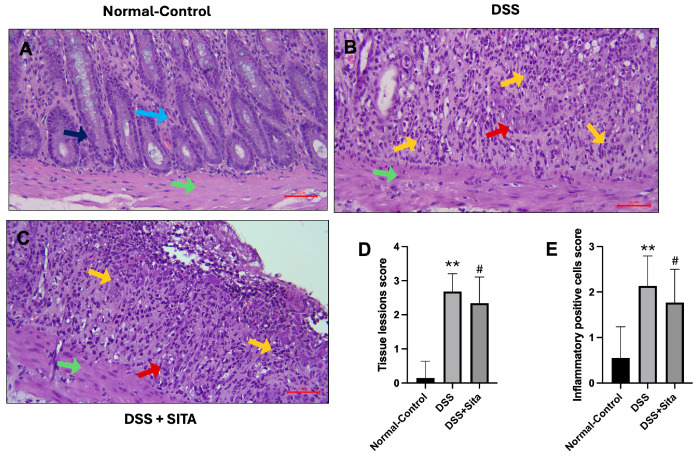
Sitagliptin improved pathological changes in DSS-induced colitis rats. (**A**) Normal-control group. (**B**) DSS group. (**C**) DSS + Sitagliptin 15 mg/kg/day group. (**D**) Tissue lesion score according to the criteria described in the method. (**E**) Inflammatory cell infiltration scores according to the criteria described in the method. The blue arrow indicated lamina propria, the green arrow indicated muscularis mucosa, the black arrow indicated normal crypt, the yellow arrow indicated infiltration of inflammatory cells, and the red arrow indicated aberrant crypt foci. Data are expressed as the mean ± SD (*n* = 6 rats/group). ** *p* < 0.01 vs. normal-control group; ^#^ *p* < 0.05 vs. DSS group. Magnification ×200. Scale bar, 50 μm.

Finally, to assess damage to the colonic mucosa and inflammatory cell infiltration, we examined the colonic tissue histopathologically. Histopathological staining and examination were conducted on colonic sections from various groups. Histological analysis revealed that DSS administration in drinking water resulted in widespread damage to the epithelial layer and significant infiltration of inflammatory cells inside the epithelium and lamina propria of the colon, with a notable increase in the histological score. The administration of sitagliptin resulted in reduced infiltration of inflammatory cells and tissue damage, significantly lowering the histological score (Figure 4A–E).

## 4. Discussion

The administration of sitagliptin in rats with DSS-induced colitis revealed a notable reduction in colitis symptoms. Physiologically, sitagliptin lessened the frequency of hematochezia, diarrhea, and weight loss. Additionally, in the colitis model induced by DSS, the administration of sitagliptin led to a decrease in the levels of MDA and the DAI score. The beneficial effect of sitagliptin on the intestinal mucosa is further illustrated by the improvement of the intestinal epithelial barrier and a decline in the number of inflammatory cells present in the intestinal mucosa. The damage to epithelial cell borders, injury to intestinal epithelial cells, and compromise of intercellular tight junctions resulted in increased permeability of the intestinal mucosa in rats treated with DSS, demonstrating that administering 5% DSS in drinking water for a duration of seven days can lead to colitis in rats, with signs similar to those observed in humans.

Oxygen free radicals significantly contribute to the development of UC and have been demonstrated to impair the intestinal mucosal barrier [5]. It has been demonstrated that in IBD, including UC, ROS may initiate lipid peroxidation within the cellular membranes of intestinal epithelial cells. This process results in the generation of lipid peroxides, such as MDA, which exhibit high reactivity and contribute to cellular damage and inflammation [18]. Moreover, the depletion of antioxidant defenses may significantly impair the inflamed mucosa of UC, increasing its vulnerability to oxygen-induced damage and hindering the recovery of the mucosa and the restoration of the epithelial cell layer’s integrity [5,19]. Previous studies have demonstrated that sitagliptin has antioxidant activity [20,21]. Consistent with previous studies, we also found that administration of sitagliptin at a dose of 15 mg/kg for 8 days in a model of experimental colitis significantly reduced MDA levels and increased levels of antioxidants, GPx, SOD, and catalase.

Previous studies have reported elevated DPP4 gene expression and enzymatic activity in inflamed colonic tissue, supporting the rationale for targeting DPP4 as a therapeutic approach to attenuate intestinal inflammation [10,22]. The inflammatory process, characterized by increased secretion of pro-inflammatory cytokines such as TNF-α and IL-1β, leads to disruption of intestinal epithelial integrity and suppresses the expression of TJ proteins, including ZO-1 and occludin [11]. Moreover, oxidative stress is also a major contributor to TJ disruption in colitis. Excessive ROS impair epithelial barrier function by disturbing the localization and stability of TJ proteins [23]. Sitagliptin has previously been demonstrated to inhibit DPP4 activity in both serum and intestinal tissue [10,22]. In addition, other DPP-4 inhibitors, such as alogliptin and saxagliptin, have also been reported to alleviate inflammation and oxidative stress and increase the expression of TJ proteins in UC [24,25]. In line with this evidence, our study demonstrated that sitagliptin, administered at a dose comparable to human therapeutic use, exerts significant anti-inflammatory and antioxidant effects in experimental colitis. This was evidenced by a marked downregulation of IL-1β and TNF-α mRNA expression, together with clear improvements in colonic histopathology and enhanced epithelial barrier integrity, as reflected by increased expression of ZO-1 and occludin.

It has recently been discovered that iron dysregulation is involved in abnormal intestinal cell death in UC, characterized by increased lipid peroxidation, depletion of glutathione peroxidase and SOD, and an imbalance in iron homeostasis [26]. In this study, we observed not only increased MDA levels and decreased GPx, SOD, and catalase levels, but also decreased serum iron levels in UC rats. These findings may reflect inflammation-associated disturbances in systemic iron homeostasis and impaired intestinal function. Restoration of serum iron levels following sitagliptin treatment may indicate an improvement in inflammatory status and intestinal integrity. Interestingly, dysregulated iron metabolism has also been increasingly associated with ferroptosis-related oxidative injury in inflammatory disorders, suggesting a potential link between iron imbalance, oxidative stress, and epithelial damage in colitis [7,24,27].

In the present study, the significant increase in SGOT/SGPT levels in UC rats compared to the group of healthy rats is most likely due to systemic inflammation and organ damage associated with oxidative stress [8]. Interestingly, sitagliptin can reduce SGOT/SGPT levels to near-normal values. This is consistent with a previous study that demonstrated that sitagliptin can improve liver function and hematologic parameters in patients with type 2 diabetes [28,29]. Collectively, these findings indicate that DPP4 inhibition may mitigate inflammatory responses, oxidative stress, iron dysregulation, and intestinal barrier dysfunction in colitis, as evidenced by the improvement of serum iron alterations, SGOT/SGPT levels, and TJ proteins. These findings further support the potential therapeutic relevance of sitagliptin in intestinal inflammatory disorders and suggest its possible protective role against systemic oxidative tissue injury associated with experimental colitis. However, caution is needed when translating these findings to clinical settings. Whether sitagliptin could reduce blood glucose levels in normoglycemic patients with colitis remains unclear, and this represents an important safety consideration. Moreover, the use of an animal model limits the generalizability of our results to human colitis, highlighting the need for further preclinical studies, such as chronic models and clinical studies. Further mechanistic studies evaluating signaling molecules such as NF-κB, Nrf2, GLP-2, and ferroptosis-related markers are required to clarify the precise molecular mechanism underlying the protective effects of sitagliptin in DSS-induced colitis. Another limitation of the present study is that gut microbiota analysis was not performed. Therefore, the potential contribution of microbiota analysis to the protective effects of sitagliptin in DSS-induced colitis could not be determined. Nevertheless, a previous study has demonstrated that sitagliptin may ameliorate microbial dysbiosis and improve intestinal TJ integrity in an experimental model of type 2 diabetes mellitus, suggesting a possible role of microbiota-related mechanisms in its intestinal protective effects [30]. Further studies incorporating microbiome profiling are warranted to better elucidate the interaction between sitagliptin, gut microbiota, intestinal barrier integrity, and colitis-associated inflammation. Moreover, incorporating a healthy sitagliptin-treated group is warranted to further clarify its basal pharmacological effects; it is also necessary to check TJ at the protein level, since mRNA levels do not always directly correlate with protein abundance or functional activity.

## 5. Conclusions

In conclusion, the present study demonstrates that sitagliptin exerts protective effects against DSS-induced colonic injury through coordinated modulation of oxidative stress, inflammation, epithelial barrier dysfunction, and alterations in iron homeostasis. The improvement of lipid peroxidation, antioxidant defense systems, intestinal TJ proteins, and serum iron levels suggests that sitagliptin may attenuate oxidative epithelial injury associated with experimental colitis. These findings expand the current understanding of the extra-glycemic properties of sitagliptin and support its potential therapeutic relevance in UC.

## Figures and Tables

**Table 1:** Primer used for qRT-PCR analysis.

Gene	Primer	Product Size (bp)
*β Actin*	F: TGT TGT CCC TGT ATG CCT CT	20
	R: TAA TGT CAC GCA CGA TTT CC	20
*ZO-1*	F: TCC ACC GGA GTC TGC TAT TA	20
	R: CTT GTG GTG AGT AAG GAG GAT ATG	24
*Occludin*	F: CGG TAC AGC AGC AAC GAT AA	20
	R: GTT TCA TAG TGG TCT GGG TCT G	22
*IL-1β*	F: CTG AAA GCT CTC CAC CTC AAT	21
	R: CGT TGC TTG TCT CTC CTT GTA	21
*TNF-α*	F: TCT ACT CCC AGG TTC TCT TCA	21
	R: CTC CTG GTA TGA AAT GGC AAA TC	23

**Table 2:** Effects of sitagliptin on the biochemical parameters in the serum.

Biochemical Parameters	Normal-Control (*n* = 6)	DSS (*n* = 6)	DSS + Sita (*n* = 6)
Fe (μg/dL)	22,357.9 ± 507	9716.8 ± 642 **	21,970.3 ± 2712 ^##^
SGOT (U/L)	45.5 ± 4.2	62.5 ± 3.1 **	53.3 ± 2.8 ^#^
SGPT (U/L)	22.9 ± 1	35.4 ± 2.5 **	32.8 ± 1 ^#^

** *p* < 0.01 vs. Normal-Control. ^#^ *p* < 0.05 vs. DSS. ^##^ *p* < 0.01 vs. DSS.

## Data Availability

All data generated or analyzed during this study are included in this article.

## Abbreviations

The following abbreviations are used in this manuscript:

DAI	Disease activity index
DPP-4	Dipeptidyl peptidase-4
DSS	Dextran sulfate sodium
GPx	Glutathione peroxidase
GLP-1	Glucagon-like peptide-1
IL-1β	Interleukin-1β
IBD	Inflammatory bowel disease
MDA	malondialdehyde
SGOT	Serum glutamic-oxaloacetic transaminase
SGPT	Serum glutamic-pyruvic transaminase
SOD	Superoxide dismutase
TBA	Thiobarbituric acid
TCA	Trichloroacetic acid
TNF-α	Tumor necrosis factor-α
UC	Ulcerative colitis
ZO-1	Zonula occludens-1
